# Portosystemic Anastomoses: A Narrative Review of Anatomy, Clinical Significance of Spontaneous Portosystemic Shunts, and Contemporary Therapeutic Approaches

**DOI:** 10.7759/cureus.115911

**Published:** 2026-09-07

**Authors:** Angelos Ntalaoutis, Theodore Troupis, Dimosthenis Chrysikos, Dimitrios Filippou

**Affiliations:** 1 2nd Department of Internal Medicine, General Hospital of Nikaia "Agios Panteleimon", Nikaia, GRC; 2 Department of Anatomy, National and Kapodistrian University of Athens, Athens, GRC

**Keywords:** brto, hepatic encephalopathy, liver cirrhosis, portal hypertension, portosystemic anastomoses, spontaneous portosystemic shunts, spss, tips, total shunt area

## Abstract

Portosystemic anastomoses represent natural or acquired vascular communications between the portal venous system and the systemic circulation, which under physiological conditions constitute vessels of minimal flow and no clinical significance, yet become dilated and develop in states of portal hypertension, predominantly in the context of liver cirrhosis. This narrative review aims to elucidate the complex and essentially dual role of these anastomoses, with particular focus on the spontaneous portosystemic shunts (SPSS) that develop in cirrhotic patients, their clinical significance, and the contemporary therapeutic approaches developed for their management. A systematic literature search was conducted across PubMed/MEDLINE, Scopus, and Google Scholar (from September 2025 to July 2026), without language restrictions, using MeSH terms and free-text keywords including "portosystemic anastomoses," "spontaneous portosystemic shunts," "portal hypertension," "hepatic encephalopathy," "TIPS," and "BRTO." A structured search strategy was applied, incorporating Boolean operators (AND/OR). No formal quantitative synthesis (meta-analysis) was performed. This is a narrative review synthesising published findings from eligible studies. A total of 847 initial records were identified, 644 were screened after duplicate removal, 123 were assessed for full-text eligibility, and 46 studies were ultimately included. The synthesis of the literature reveals that portosystemic anastomoses are anatomically classified into oesophagogastric, anorectal, paraumbilical, and retroperitoneal types, with the spontaneous splenorenal shunt (SSRS) representing the most commonly identified large SPSS in advanced cirrhosis. The presence of SPSS, particularly when their total shunt area (TSA) exceeds 83 mm², is independently associated with an increased risk of hepatic encephalopathy (HE) and mortality, regardless of the Child-Pugh or Model for End-stage Liver Disease (MELD) score. The relationship between SPSS and variceal bleeding is complex and counterintuitive, as the presence of SPSS does not appear to confer protection against haemorrhagic events. Therapeutic management relies primarily on the transjugular intrahepatic portosystemic shunt (TIPS) and on selective embolisation techniques, including balloon-occluded retrograde transvenous obliteration (BRTO), plug-assisted retrograde transvenous obliteration (PARTO), and coil-assisted retrograde transvenous obliteration (CARTO), while surgical shunts retain a role in selected patient categories. This review concludes that portosystemic anastomoses are not a neutral imaging finding but an active participant in the natural course of cirrhosis, with an initially compensatory yet ultimately detrimental character. Their recognition, quantification, and individualised management are critical parameters in the contemporary clinical approach to the patient with cirrhosis and portal hypertension.

## Introduction and background

The portal venous system carries nutrient-rich blood from the gastrointestinal tract, spleen, and pancreas to the liver, contributing 70-75% of total hepatic blood flow. Portal hypertension is defined as an elevation of the hepatic venous pressure gradient (HVPG) to ≥6 mmHg and represents the final common pathway of chronic liver disease, predominantly cirrhosis. In Western countries, cirrhosis accounts for more than 90% of portal hypertension cases. The full aetiological classification is presented in Table 1. Clinically significant portal hypertension (CSPH) is defined as HVPG ≥10 mmHg, with the ≥12 mmHg threshold corresponding to the risk of variceal bleeding and decompensation (Figure 1) [1-4].

**Table 1:** Aetiological classification of portal hypertension. HVPG: hepatic venous pressure gradient; NAFLD: non-alcoholic fatty liver disease; NASH: non-alcoholic steatohepatitis. ^*^Schistosomiasis: the most common non-cirrhotic cause worldwide. *Source*: Original table created by the authors based on reference [4].

Category	Type	Causes	HVPG
Prehepatic	Extrahepatic obstruction	Portal/splenic vein thrombosis, arteriovenous fistula, external compression	Normal
Intrahepatic	Presinusoidal	Schistosomiasis*, primary biliary cholangitis, hepatoportal vasculopathy, granulomatous diseases	Normal
	Sinusoidal	Cirrhosis (most common), acute alcoholic hepatitis, NAFLD/NASH, hepatocellular carcinoma	Elevated
	Postsinusoidal	Veno-occlusive disease, alcoholic cirrhosis (partial)	Elevated
Posthepatic	Extrahepatic venous obstruction	Budd-Chiari syndrome, right heart failure, constrictive pericarditis, inferior vena cava thrombosis	Elevated

**Figure 1:**
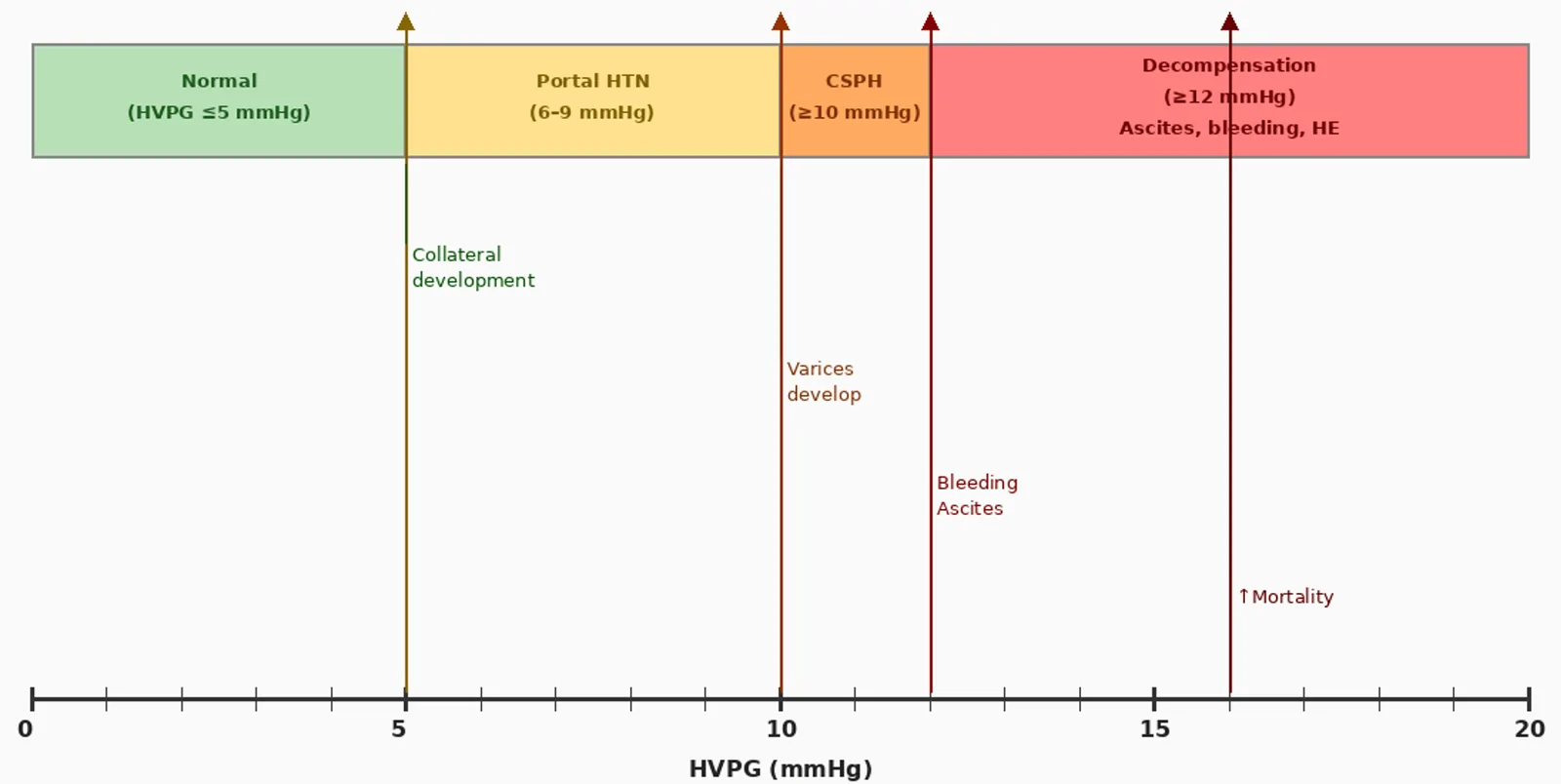
Clinical significance of the HVPG in portal hypertension. HVPG: hepatic venous pressure gradient; CSPH: clinically significant portal hypertension; HE: hepatic encephalopathy; HTN: hypertension. *Source*: Original figure created by the authors based on reference [4].

Sustained elevation of portal pressure drives the development of portosystemic collateral pathways through a combination of passive dilation of pre-existing vascular communications and active neoangiogenesis mediated by vascular endothelial growth factor (VEGF). These collaterals, portosystemic anastomoses, form at predictable anatomical sites where portal and systemic venous territories communicate: the oesophagogastric junction, the anorectal plexus, the paraumbilical region, and the retroperitoneum (Figure 2) [1,2,5].

**Figure 2:**
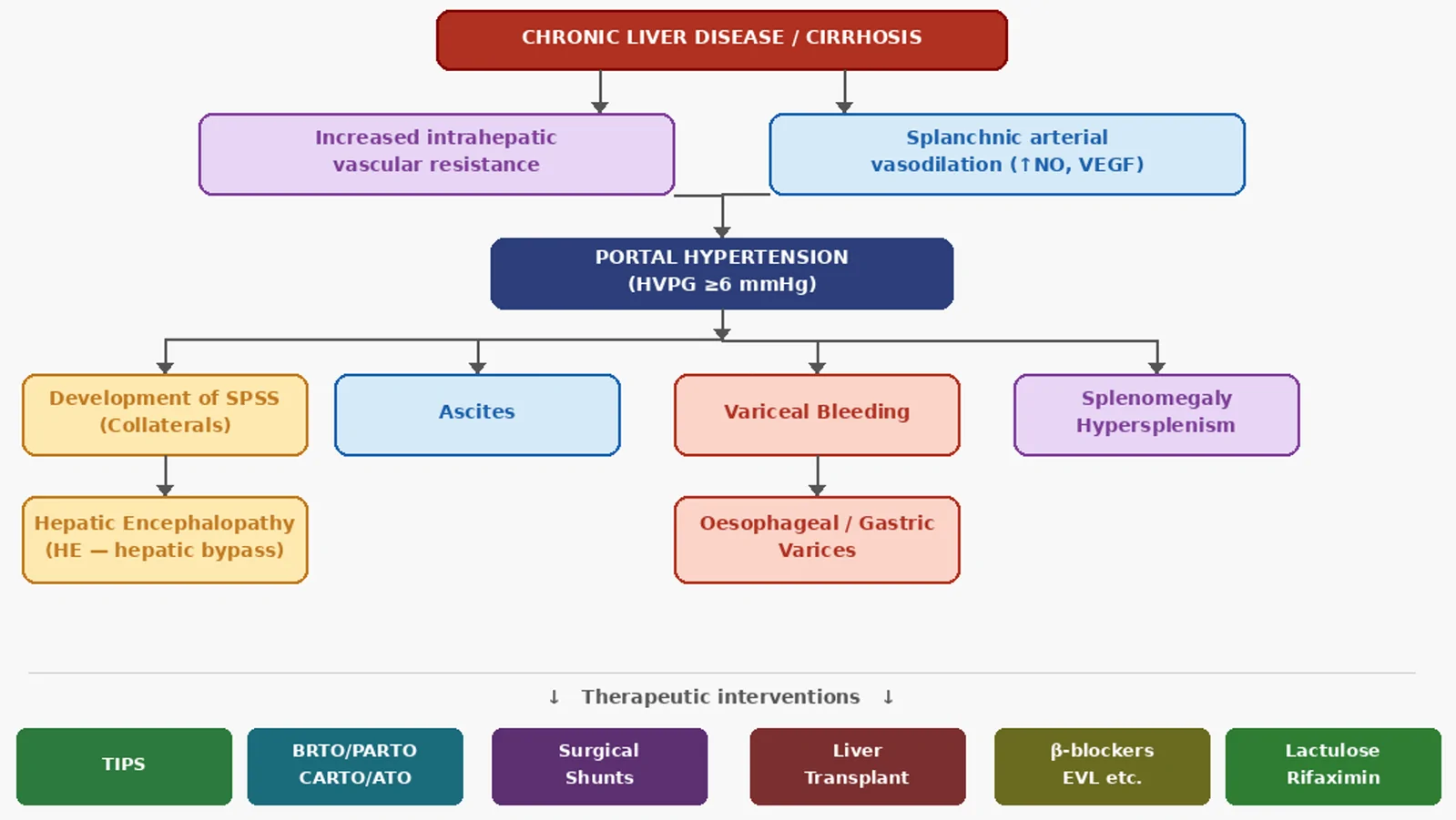
Pathophysiology of portal hypertension and portosystemic anastomoses. SPSS: spontaneous portosystemic shunts; HE: hepatic encephalopathy; NO: nitric oxide; VEGF: vascular endothelial growth factor; HVPG: hepatic venous pressure gradient; TIPS: transjugular intrahepatic portosystemic shunt; BRTO: balloon-occluded retrograde transvenous obliteration; PARTO: plug-assisted retrograde transvenous obliteration; CARTO: coil-assisted retrograde transvenous obliteration; ATO: anterograde transvenous obliteration; EVL: endoscopic variceal ligation. *Source*: Original figure created by the authors based on reference [5].

Among these collaterals, spontaneous portosystemic shunts (SPSS), particularly large extrahepatic shunts such as the spontaneous splenorenal shunt (SSRS) and the recanalized paraumbilical vein (rPUV), have emerged as clinically important independent determinants of outcome in cirrhosis. Their presence reflects both the severity of portal hypertension and an active adaptive response that, paradoxically, may become maladaptive over time. While initially decompressing the portal circulation, large SPSS increasingly divert portal blood away from the liver, contributing to hepatic encephalopathy (HE), impaired hepatic synthetic function, and adverse prognosis [6-8].

The management of portal hypertension and its complications, including variceal bleeding, refractory ascites, and HE, has been transformed by the introduction of the transjugular intrahepatic portosystemic shunt (TIPS), the development of endoscopic and interventional radiology techniques, and the evolution of international consensus guidelines, including Baveno VII, the 2024 AASLD Practice Guidance, the 2025 EASL Clinical Practice Guidelines on TIPS, and the 2026 EASL Clinical Practice Guidelines on vascular diseases of the liver [4,9-11]. The specific management of large SPSS, including the decision between TIPS, targeted embolisation, or combined approaches, has attracted increasing attention as a distinct clinical challenge [8,12,13].

This narrative review addresses the following knowledge gap: the dual (initially compensatory and ultimately detrimental) role of portosystemic anastomoses in cirrhosis, with a focus on SPSS anatomy and classification, imaging and quantification, clinical consequences and prognostic significance, and contemporary therapeutic decision-making. No formal quantitative synthesis was performed given the heterogeneity of available studies; instead, a structured narrative approach was used to evaluate and integrate the available evidence, with explicit identification of evidence limitations.

## Review

Literature search methodology

A structured literature search was conducted across PubMed/MEDLINE, Scopus, and Google Scholar, covering the period from September 2025 to July 2026, without language restrictions. PubMed/MEDLINE was searched using MeSH terms: "portosystemic shunt, surgical," "hypertension, portal," "hepatic encephalopathy," combined with free-text keywords: "spontaneous portosystemic shunt," "SPSS," "total shunt area," "TSA," "BRTO," "PARTO," "CARTO," "TIPS," AND "portal hypertension." Boolean operators AND/OR were applied across all databases. Search limits included: clinical studies, reviews, meta-analyses, and guidelines published from 2000 onwards, with priority given to publications from 2015 to 2026.

Inclusion Criteria

Studies reporting outcomes of SPSS (prevalence, clinical impact, treatment) or portal hypertension management in adult patients with cirrhosis or non-cirrhotic portal hypertension; international clinical practice guidelines relevant to the topic.

Exclusion Criteria

Case reports with n<5, non-peer-reviewed sources, and non-English abstracts without an available translation. No formal risk-of-bias assessment was conducted, consistent with the narrative review design; however, study quality and evidence level are discussed where relevant. No quantitative synthesis (meta-analysis) was performed; findings are synthesised through narrative methods. This review is explicitly a narrative, not a systematic, review; the structured/PRISMA-inspired search approach was used for transparency of the literature search process, without implying formal PRISMA compliance. Of 847 initial records identified, 644 were screened after duplicate removal, 123 were assessed for full-text eligibility, and 46 studies were ultimately included.

Anatomy and classification of portosystemic anastomoses

Portosystemic anastomoses are vascular communications between the portal venous system and the systemic venous circulation through which blood bypasses the liver. Under physiological conditions, they exist as latent vessels; in portal hypertension, they dilate and develop as clinically significant collateral pathways [14,15].

Oes​​ophagogastric Anastomoses

These are the most common and clinically significant type, occurring in 80-90% of patients with portal hypertension. The left gastric vein reverses flow in portal hypertension, draining via the submucosal plexus of the distal oesophagus into the azygos and hemiazygos veins. Gastric varices are classified by the Sarin classification into gastroesophageal varices (GOV1 and GOV2) and isolated gastric varices (IGV1 and IGV2). IGV1 in the gastric fundus are frequently associated with SSRS and carry a higher haemorrhagic risk [16,17].

Anorectal Anastomoses

The superior rectal vein (inferior mesenteric vein tributary) communicates with the middle and inferior rectal veins (internal iliac system). Anorectal varices occur in 44-78% of cirrhotic patients and must be distinguished from haemorrhoids [5,17].

Paraumbilical Anastomoses

Paraumbilical veins (remnants of the foetal umbilical system) connect the left portal vein branch to the anterior abdominal wall epigastric veins, producing the characteristic caput medusae. A rPUV is among the most frequently identified SPSS on computed tomography (CT) imaging [14,17-19].

Retroperitoneal Anastomoses

Collaterals form between mesenteric veins and renal, adrenal, or iliac veins. The SSRS (the most clinically important large SPSS) diverts blood from the splenic vein to the left renal vein and subsequently to the inferior vena cava [6,17,20].

Ectopic Anastomoses

These include cholecystic, pancreaticoduodenal, adrenal, and gonadal collaterals. Ectopic varices occur in 1-5% of cirrhotic patients and may bleed unpredictably [17,21].

SPSS: definition, pathogenesis, and classification

Definition and Distinction From Congenital Shunts

SPSS are acquired vascular communications developing in portal hypertension through dilation of pre-existing vascular networks or neoangiogenesis [22]. They are distinct from congenital portosystemic shunts (Abernethy malformation), which exist from birth independently of portal hypertension [23-26].

Pathogenesis

The primary mechanism is elevated portal pressure creating a haemodynamic gradient driving blood toward low-resistance pathways. VEGF-mediated neoangiogenesis plays a key regulatory role [5,14]. SPSS evolution is dynamic: Vidal-González et al. documented progressive enlargement with worsening cirrhosis and regression following aetiological intervention (sustained virological response and alcohol abstinence) [27].

Classification and Prevalence

The Baveno VI-SPSS group classified SPSS into those within the oesophagogastric region (oesophageal and gastric varices) and those outside it (SSRS, rPUV, and ectopic varices). Extra-oesophagogastric SPSS are more strongly associated with HE, while oesophagogastric varices remain the predominant source of variceal bleeding (Table 2) [6,7,21].

**Table 2:** Comparison of main SPSS types. SSRS: spontaneous splenorenal shunt; IGV1: isolated gastric varices type 1; rPUV: recanalized paraumbilical vein; TSA: total shunt area; TIPS: transjugular intrahepatic portosystemic shunt; BRTO: balloon-occluded retrograde transvenous obliteration; PARTO: plug-assisted retrograde transvenous obliteration; ATO: anterograde transvenous obliteration; HE: hepatic encephalopathy. *Source*: Original table created by the authors based on references [6,16,17].

SPSS type	Anatomy	Clinical significance	Preferred treatment
Splenorenal (SSRS)	Splenic vein → left renal vein	Most common large SPSS, strongly associated with HE, highest risk of decompensation	BRTO/PARTO or TIPS + embolisation
Gastrorenal	Short gastric veins → renal vein	IGV1, high bleeding risk, massive haemorrhage is possible	BRTO—first line
Paraumbilical (rPUV)	Left portal branch → epigastric veins	Caput medusae, usually lower clinical impact, common computed tomography finding	Embolisation if TSA >83 mm²
Splenocaval	Splenic vein → inferior vena cava	Rare, HE if large shunt	Embolisation
Ectopic varices	Mesenteric veins → iliac/renal veins	Unpredictable bleeding, difficult to manage endoscopically	ATO or TIPS + embolisation

In the study by Nardelli et al. using CT, SPSS were detected in 49% of patients with cirrhosis [19]. SSRS occurs in 15-25% of patients with cirrhosis, whereas rPUV occurs in 20-30%. In advanced cirrhosis (Child-Pugh B/C), overall SPSS prevalence exceeds 60-70% [6,7,17].

Imaging and quantification of SPSS

Triphasic abdominal CT is the reference standard for SPSS mapping, enabling anatomical characterisation and measurement of the total shunt area (TSA), the sum of the cross-sectional areas of all identified SPSS, expressed as mm². A TSA > 83 mm² has been established as a clinically meaningful threshold independently predicting overt HE and mortality (Figure 1) [7,19]. Magnetic resonance imaging (MRI) offers excellent vascular imaging without radiation and is particularly useful in patients with contraindications to iodinated contrast. Doppler ultrasonography is the first-line tool for portal patency assessment but has limited sensitivity for small or retroperitoneal SPSS [8,20].

Clinical consequences of SPSS

The clinical importance of SPSS derives from their dual role: initially compensatory (reducing portal pressure), but ultimately detrimental, as they divert portal blood away from the liver, bypassing hepatic detoxification and reducing hepatic perfusion [7,8].

SPSS and HE

The association between SPSS and HE is the most robustly documented in the literature. Diversion of ammonia and other neurotoxins from gut to systemic circulation, bypassing hepatic detoxification, is the primary mechanism [12,28]. In the landmark multicentre study by Praktiknjo et al. (n=908, 11 centres), TSA >83 mm² independently predicted overt HE (hazard ratio [HR] 1.7; 95% confidence interval [CI] not reported in the original study) and mortality, irrespective of Child-Pugh or MELD scores [7]. Critically, this association was independent of conventional prognostic scores, highlighting the additional value of TSA measurement. The Baveno VI-SPSS study by Simón-Talero et al. (n=1,020) confirmed that extra-oesophagogastric SPSS increase the risk of HE two- to threefold [6].

Regarding the management of shunt-induced HE, the meta-analysis by Wang et al. (10 retrospective studies, n=289 patients) reported that SPSS embolisation achieved HE remission in 83.1% (95% CI: 70.4%-93.1%), with a procedural success rate of 99.8% (95% CI: 98.3%-100%) and long-term adverse event rate of 42.9% (95% CI: 34.7%-51.4%; I² not reported) [29]. A multicentre series by Laleman et al. (n=37, retrospective) reported 77% HE remission [30]. These figures should be interpreted in the context of the predominantly retrospective and heterogeneous nature of the underlying data, with significant inter-study variability in patient selection, shunt anatomy, and technical approach.

SPSS and Variceal Bleeding

Counterintuitively, the presence of SPSS does not protect against variceal bleeding. Simón-Talero et al. demonstrated that patients with extra-oesophagogastric SPSS did not have a reduced variceal bleeding risk, and some subgroups showed an increased risk [6]. A 2024 review confirmed that recent data suggest that SPSS increases the risk of variceal bleeding regardless of shunt size [8]. IGV1 gastric varices, frequently associated with SSRS, carry a higher risk of haemorrhage than oesophageal varices due to maintained high pressures in the gastric fundal circulation [16].

SPSS and Ascites

Large SPSS reduce portal pressure but simultaneously reduce hepatic perfusion, accelerating hepatocyte dysfunction and worsening albumin synthesis and renal perfusion. Furthermore, SPSS embolisation for HE may precipitate or worsen ascites through an acute elevation in portal pressure [6,12,14,30].

SPSS and Hepatic Function

Rathi et al. demonstrated that patients with SPSS had significantly lower albumin levels and higher ammonia concentrations compared to patients with equivalent liver disease severity without SPSS, confirming that large SPSS independently impair hepatic synthetic and detoxifying function [20].

Prognostic significance of SPSS

SPSS constitute an independent adverse prognostic factor beyond MELD and Child-Pugh. The Baveno VI-SPSS study showed significantly higher rates of HE, hospitalisation, and mortality over two years in patients with SPSS [6]. García-Villarreal et al. confirmed that massive SPSS (diameter >10 mm) are easily identifiable independent prognostic markers [31]. Dajti et al. demonstrated that SSRS location, independent of size, confers the highest risk of decompensation among SPSS types [32]. Hintersteininger et al. demonstrated that TIPS placement reduces TSA over time, with a reduction in TSA correlating with improved prognosis [33].

Therapeutic management: TIPS

Historical Development and Technique

TIPS was developed experimentally in the 1960s and entered clinical use in the late 1980s following the first successful stent-based procedures. The introduction of expanded polytetrafluoroethylene (ePTFE)-covered stents reduced stent dysfunction rates to less than 20% at two years, compared to approximately 50% with bare-metal stents [34,35]. TIPS creates an intrahepatic communication between a hepatic vein and a portal vein branch via transjugular access, using an 8-10 mm Viatorr covered stent, targeting HVPG <12 mmHg or portosystemic pressure gradient (PSPG) reduction of ≥20% from baseline [16,36].

Indications

Current international guidelines, including the 2025 EASL Clinical Practice Guidelines on TIPS, the 2024 AASLD Practice Guidance, and the Baveno VII consensus, recommend TIPS for the following indications: (1) Refractory/recurrent ascites, Bureau et al. (randomised controlled trial (RCT), n=62) demonstrated significant survival benefit over large-volume paracentesis (LVP) [13,37]; (2) pre-emptive TIPS within 72 hours for high-risk variceal bleeding (Child-Pugh B with active bleeding or Child-Pugh C 10-13), García-Pagán et al. (RCT, n=63) demonstrated mortality reduction from 33% to 14% [38,39]; (3) secondary variceal bleeding prophylaxis after endoscopic failure; (4) hepatic hydrothorax; (5) selected non-cirrhotic portal hypertension and vascular liver diseases as per the 2026 EASL vascular liver disease guidelines [11,40].

Contraindications

Absolute contraindications include severe heart failure, uncontrolled sepsis, and polycystic liver, whereas relative contraindications include Child-Pugh >13, MELD >18-20, Grade ≥2 HE, and central hepatocellular carcinoma [16,36,38].

Complications

HE is the most common complication, occurring in 25-45% of patients within one year [41]; stent dysfunction in 10-20% of patients with covered stents [35]. Larrue et al. (individual patient data meta-analysis, n=1,017 patients from 4 RCTs) demonstrated that early TIPS prevents further decompensation and improves transplant-free survival (HR 0.60; 95% CI 0.42-0.85) [42]. TIPS placement in patients with pre-existing large SPSS carries additional HE risk due to cumulative portal flow diversion, a finding that supports combined TIPS + SPSS embolisation strategies in selected patients [13,33]. In a landmark study, García-Pagán et al. demonstrated that pre-emptive TIPS markedly reduced further decompensation and mortality [39].

Therapeutic management: SPSS embolisation

Selective embolisation of large SPSS targets the shunt directly, aiming to redirect portal blood back toward the liver. The 2025 EASL TIPS guidelines and the AASLD 2024 guidance explicitly address SPSS embolisation as an adjunct or alternative to TIPS in selected patients (Table 3) [13,16].

**Table 3:** Comparative assessment of TIPS versus SPSS embolisation. TIPS: transjugular intrahepatic portosystemic shunt;  SPSS: spontaneous portosystemic shunt; HE: hepatic encephalopathy; SSRS: spontaneous splenorenal shunt; HVPG: hepatic venous pressure gradient; PSPG: portosystemic pressure gradient; TSA: total shunt area; RCT: randomised controlled trial; BRTO: balloon-occluded retrograde transvenous obliteration; PARTO: plug-assisted retrograde transvenous obliteration; CARTO: coil-assisted retrograde transvenous obliteration; ATO: anterograde transvenous obliteration. *Source*: Original table created by the authors based on references [29,30,33,37,39,42].

Parameter	TIPS	Embolisation (BRTO/PARTO/CARTO/ATO)
Mechanism	Controlled diversion of portal flow (artificial shunt)	Occlusion of a natural SPSS → portal pressure elevation
Main indication	Variceal bleeding; refractory ascites; pre-emptive TIPS	Shunt-induced HE; gastric varices with SSRS
Effect on HE	Risk of de novo or worsened HE (25–45% within one year)	HE remission 77–83% (Wang et al. [29], Laleman et al. [30])
Effect on portal pressure	Reduces HVPG/PSPG; target <12 mmHg	Increases PSPG → variceal or ascites risk (33%)
Effect on SPSS	Reduces TSA long-term (Hintersteininger et al. [33])	Occludes the targeted SPSS
Reversibility	Potentially reversible (reduction stent)	Irreversible
Evidence quality	Multiple RCTs (Bureau et al. [37]; García-Pagán et al. [39]; Larrue et al. [42])	Mainly retrospective data (no RCT vs. TIPS)
Geographic preference	Europe/North America	Japan/South Korea (especially BRTO)

BRTO, PARTO, CARTO, and ATO

BRTO (balloon-occluded retrograde transvenous obliteration), developed in Japan in the 1980s, occludes the draining vein of the SPSS while a sclerosant is injected. Primarily indicated for SSRS with IGV1 gastric varices or shunt-induced HE [12,16]. PARTO (plug-assisted retrograde transvenous obliteration) uses a vascular plug instead of a balloon, enabling faster completion. CARTO (coil-assisted retrograde transvenous obliteration) employs embolisation coils. ATO (anterograde transvenous obliteration) is performed via transhepatic or transsplenic access when retrograde access is anatomically unfeasible [12]. Selection among techniques depends on SPSS anatomy, material availability, and centre expertise.

Therapeutic management: surgical portosystemic shunts

Surgical shunts have been largely supplanted by TIPS in most cirrhotic indications but retain a role in selected patients, particularly in non-cirrhotic portal hypertension [43].

Types of Surgical Shunts

The portocaval shunt constitutes the original surgical procedure [34,44]; the mesocaval shunt connects the superior mesenteric vein to the inferior vena cava via an ePTFE graft [43,44]; the central splenorenal shunt anastomoses the splenic stump to the left renal vein. The distal splenorenal shunt (DSRS) selectively decompresses the oesophagogastric zone while maintaining mesenteric portal flow to the liver, resulting in lower HE rates compared to total shunts [43,45,46]. Per the 2026 EASL vascular liver disease guidelines, surgical shunts may be considered in selected patients with non-cirrhotic portal hypertension when TIPS is unavailable [40].

Discussion

The Dual Role of SPSS: Compensatory and Detrimental Effects

Historically regarded as protective collaterals, SPSS are now recognised as having a dual role determined by size, location, and stage of cirrhosis. At the critical TSA threshold of >83 mm², hepatic bypass outweighs the benefit of portal pressure reduction [6,7,20,32]. Small SPSS in compensated cirrhosis may be clinically neutral; large SSRS in decompensated cirrhosis constitute an independent mortality predictor beyond MELD and Child-Pugh [31]. Importantly, observational associations between SPSS and adverse outcomes should not be interpreted as causal in the absence of randomised data.

TIPS Versus SPSS Embolisation: A Clinical Decision Framework

TIPS and SPSS embolisation represent fundamentally different approaches (controllable versus targeted) to portal decompression. The 2025 EASL TIPS guidelines provide a framework for individualised decision-making [13]. Hintersteininger et al. documented that TIPS reduces TSA long-term [33], suggesting TIPS may treat SPSS themselves. In patients with large pre-existing SPSS, cumulative portal diversion from TIPS carries additional HE risk, supporting combined TIPS + SPSS embolisation strategies [12,30,42].

The choice between approaches depends on (1) the type of complication: variceal bleeding and refractory ascites favour TIPS; shunt-induced HE with large SSRS may favour embolisation or combination; (2) SPSS anatomy; (3) hepatic function (Child-Pugh/MELD); (4) centre expertise and available techniques; and (5) applicable guidelines (EASL 2025 TIPS, AASLD 2024, Baveno VII) (Table 3, Figure 3) [11,13,16].

**Figure 3:**
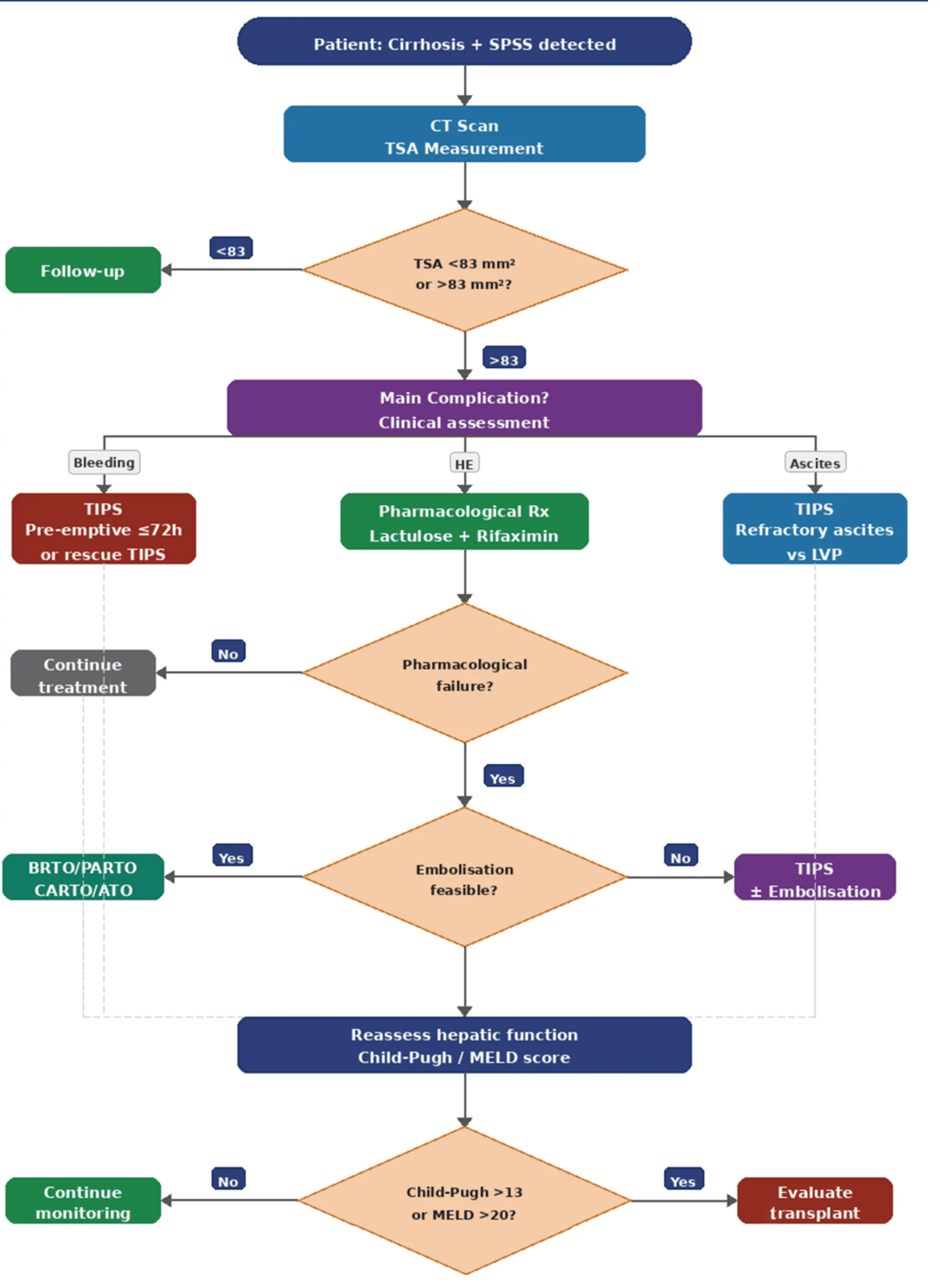
Proposed therapeutic algorithm for patients with cirrhosis and large SPSS. SPSS: spontaneous portosystemic shunts; TIPS: transjugular intrahepatic portosystemic shunt; CT: computed tomography; TSA: total shunt area; HE: hepatic encephalopathy; LVP: large-volume paracentesis; MELD: Model for End-stage Liver Disease; BRTO: balloon-occluded retrograde transvenous obliteration; PARTO: plug-assisted retrograde transvenous obliteration; CARTO: coil-assisted retrograde transvenous obliteration; ATO: anterograde transvenous obliteration. *Source*: Original figure created by the authors.

Critical Appraisal of the Literature and Evidence Limitations

A critical limitation is the absence of RCTs directly comparing TIPS and SPSS embolisation for shunt-induced HE. Available embolisation data derive predominantly from retrospective series with heterogeneous populations, variable SPSS definitions (TSA vs. diameter), and different imaging modalities, limiting inter-study comparability [12,16,29].

Significant methodological heterogeneity exists: Praktiknjo et al. [7] used TSA with an 83 mm² threshold, while García-Villarreal et al. [31] used a diameter of >10 mm, criteria that are not invariably equivalent. No study has performed formal individual-study risk-of-bias assessment. Meta-analytic HE remission estimates should be interpreted with caution given the retrospective, single-arm design of the included studies and the absence of reported I² heterogeneity statistics [29].

Geographic asymmetry warrants attention: the majority of embolisation data originates from Asian centres (Japan and South Korea), where SSRS-IGV1 prevalence and disease aetiology (hepatitis B predominant) differ substantially from Western populations (alcohol/metabolic aetiology predominant) [12,16]. Direct applicability to European patients therefore requires caution, a concern also acknowledged in the 2025 EASL TIPS guidelines [13].

Future Perspectives

Key future directions include: (1) RCTs comparing TIPS, embolisation, and combination strategies in patients with large SSRS and shunt-induced HE [16,29]; (2) incorporation of TSA into routine clinical stratification and international guidelines [7,11]; (3) prospective evaluation of prophylactic SPSS embolisation in compensated patients with TSA >83 mm² [12,27]; (4) standardisation of TSA measurement methodology across centres; (5) pharmacological targeting of VEGF-mediated neoangiogenesis [5,27]; and (6) integration of artificial intelligence for automated TSA measurement and CSPH prediction. The progressive evolution of the Baveno guidelines (I-VII) and the new EASL-dedicated TIPS and vascular liver disease guidelines reflect a paradigm shift toward holistic decompensation prevention [11,13,40,47].

## Conclusions

SPSS are not a neutral imaging finding but an active biological mechanism with substantial impact on the course of cirrhosis. Their role is dual: initially compensatory, diverting portal flow and delaying decompensation, and ultimately detrimental as their enlargement bypasses hepatic detoxification, accelerating progression to HE, impaired hepatic function, and adverse prognosis. The TSA, measured by CT, is the most reliable quantitative prognostic indicator, a TSA exceeding 83 mm² independently predicts overt HE and mortality beyond the Child-Pugh and MELD scores, warranting integration into routine clinical evaluation. The SSRS location confers the highest decompensation risk among SPSS subtypes.

Therapeutic decision-making must be individualised, as each intervention carries inherent trade-offs: SPSS embolisation (BRTO, PARTO, CARTO, ATO) effectively treats shunt-induced HE but elevates portal pressure, while TIPS reduces portal pressure but carries its own HE risk when large SPSS are already present. The 2025 EASL Clinical Practice Guidelines on TIPS and the 2026 EASL guidelines on vascular liver diseases provide a framework for this decision. Surgical shunts remain effective for selected patients with non-cirrhotic portal hypertension. Significant knowledge gaps persist: no RCT has directly compared TIPS and SPSS embolisation for shunt-induced HE, and standardised TSA measurement protocols remain to be established. The progressive evolution of consensus from Baveno I to Baveno VII, together with the new dedicated EASL guidelines, reflects a paradigm shift toward holistic decompensation prevention, with SPSS increasingly recognised as independent contributors to disease progression.
